# Use of Electronic Health Records and Geographic Information Systems in Public Health Surveillance of Type 2 Diabetes: A Feasibility Study

**DOI:** 10.2196/publichealth.4319

**Published:** 2016-03-17

**Authors:** Liliana Laranjo, David Rodrigues, Ana Marta Pereira, Rogério T Ribeiro, José Manuel Boavida

**Affiliations:** ^1^Centro de Investigação em Saúde Pública, Escola Nacional de Saúde PúblicaPortuguese School of Public HealthUniversidade Nova de LisboaLisboaPortugal; ^2^Centre for Health InformaticsAustralian Institute of Health InnovationMacquarie UniversitySydneyAustralia; ^3^NOVA Medical School/ Faculdade de Ciências MédicasFamily Medicine DepartmentUniversidade Nova de LisboaLisboaPortugal; ^4^Faculty of Human and Social SciencesUniversidade Nova de LisboaLisboaPortugal; ^5^APDP-DiabetesEducation and Research CenterUniversidade Nova de LisboaLisboaPortugal

**Keywords:** electronic health records, diabetes mellitus, geographic information systems, primary health care, health records, personal

## Abstract

**Background:**

Data routinely collected in electronic health records (EHRs) offer a unique opportunity to monitor chronic health conditions in real-time. Geographic information systems (GIS) may be an important complement in the analysis of those data.

**Objective:**

The aim of this study was to explore the feasibility of using primary care EHRs and GIS for population care management and public health surveillance of chronic conditions, in Portugal. Specifically, type 2 diabetes was chosen as a case study, and we aimed to map its prevalence and the presence of comorbidities, as well as to identify possible populations at risk for cardiovascular complications.

**Methods:**

Cross-sectional study using individual-level data from 514 primary care centers, collected from three different types of EHRs. Data were obtained on adult patients with type 2 diabetes (identified by the International Classification of Primary Care [ICPC-2] code, T90, in the problems list). GISs were used for mapping the prevalence of diabetes and comorbidities (hypertension, dyslipidemia, and obesity) by parish, in the region of Lisbon and Tagus Valley. Descriptive statistics and multivariate logistic regression were used for data analysis.

**Results:**

We identified 205,068 individuals with the diagnosis of type 2 diabetes, corresponding to a prevalence of 5.6% (205,068/3,659,868) in the study population. The mean age of these patients was 67.5 years, and hypertension was present in 71% (144,938/205,068) of all individuals. There was considerable variation in diagnosed comorbidities across parishes. Diabetes patients with concomitant hypertension or dyslipidemia showed higher odds of having been diagnosed with cardiovascular complications, when adjusting for age and gender (hypertension odds ratio [OR] 2.16, confidence interval [CI] 2.10-2.22; dyslipidemia OR 1.57, CI 1.54-1.60).

**Conclusions:**

Individual-level data from EHRs may play an important role in chronic disease surveillance, namely through the use of GIS. Promoting the quality and comprehensiveness of data, namely through patient involvement in their medical records, is crucial to enhance the feasibility and usefulness of this approach.

## Introduction

Nowadays, data collected by health care providers in electronic health records (EHRs) offer a unique opportunity to monitor acute and chronic health conditions in real-time [1]. Moreover, EHRs have the potential to become a cost-efficient, feasible, and sustainable source of data for continuous population health management [1]. One interesting way to analyze EHR-collected data is with the use of geographic information systems (GIS). GIS can track regional changes in disease incidence and prevalence, analyze the environmental and social determinants of health, identify health trends in local communities, and help plan interventions for populations with the greatest need of services [2].

GIS have the ability to give geographic context to EHR data and seem useful when conducting community-level health needs assessment. Indeed, geovisualization may be considered a preliminary stage in focusing public health efforts in high-need communities. Moreover, GIS are gathering increasing attention in the identification and analysis of high-risk areas for noncommunicable diseases, as is the case with “obesogenic environments” [3,4] and diabetes [2,5-7]. 

The main objective of this study was to evaluate the feasibility of using primary care individual-level EHR data and GIS, for public health surveillance of type 2 diabetes in Portugal.

## Methods

### Setting

This study was based in Lisbon and in the neighboring region of Tagus Valley, involving a total of 514 primary care centers. All centers were computerized and had an EHR in use. At the time of the study, there were three different types of EHR software in use at the primary care system, but one of them (named ‘SAM’, developed and funded by the Ministry of Health) was used in the great majority of primary care practices throughout the country. Data from primary care EHRs are currently gathered in local health data warehouses, one of which is in Lisbon.

In Portugal, health care is mostly publicly funded, and the majority of patients has a unique patient identifier and access to primary care services through the public primary care system. Use of the International Classification of Primary Care (ICPC-2) is common practice by primary care physicians in Portugal, especially for registering diagnoses and health problems in the EHR. Furthermore, current quality improvement indicators defined by the Ministry of Health are mostly dependent on the use of this classification.

### Data Collection

Data collection was performed in September 2013 by the Information Technology department of the Regional Health Administration in Lisbon, from its data warehouse. The dataset provided was de-identified (a pseudonymised identifier was used for each patient). Individual-level data were collected on adult patients (≥20 years of age) with the diagnosis of type 2 diabetes (identified by having the ICPC-2 code for type 2 diabetes - T90 - in the EHR field ‘problems list’). Duplicates were removed from the dataset, as well as patients living outside of the study area of Lisbon and Tagus Valley. Variables collected were: age, gender, parish of residence, comorbidities, and cardiovascular complications. Data on comorbidities and cardiovascular complications were collected from the problems list, by the presence or absence of the corresponding ICPC-2 codes (comorbidities: obesity, T82; hypertension, K86 and K87; and dyslipidemia, T93; and complications: ischemic heart disease, K74 and K76; myocardial infarction, K75; transient cerebral ischemia, K89; stroke, K90; cerebrovascular disease, K91; and peripheral vascular disease, K92).

The study was approved by the National Data Protection Committee and by the Ethics Committee of the Regional Health Administration in Lisbon.

### Data Analysis

R Studio software (version 3.0.2) was used for the statistical analyses. The ArcMap functionality of ArcGis (version 10; ESRI) was used to create cloropleth maps. The prevalence of diabetes by parish was mapped using a gray scale where the darkest tone represented the highest prevalence. The same method was applied to generate the comorbidities’ maps.

## Results

### Diabetes Prevalence

From a total of 3,659,868 individual records of people registered in the primary care centers studied, 205,068 had the diagnosis of type 2 diabetes, corresponding to a prevalence of 5.6% (205,068/3,659,868). The mean age of these patients was 67.5 years (standard deviation 11.7) and 49.8% (102,155/205,068) were female. The majority (190,912/205,068, 93.1%) of patients were 50 years of age or older.

Hypertension was present in 71% (144,938/205,068) of the patients with type 2 diabetes, obesity in 20% (41,473/205,068), and dyslipidemia in 45% (92,000/205,068); 19% (37,949/205,068) of the patients had none of these comorbidities. No cardiovascular complications were registered for 85% (173,227/205,068) of the patients. Ischemic heart disease was the most prevalent cardiovascular complication, being present in 7% of the patients (14,982/205,068), followed by stroke (9,152/205,068, 5%), peripheral vascular disease (7,683/205,068, 4%), and myocardial infarction (5,012/205,068, 2%). Transient cerebral ischemia and cerebrovascular disease were registered in less than 2% of the patients with diabetes (1,355/205,068 and 2,448/205,068, respectively).

**Figure 1:**
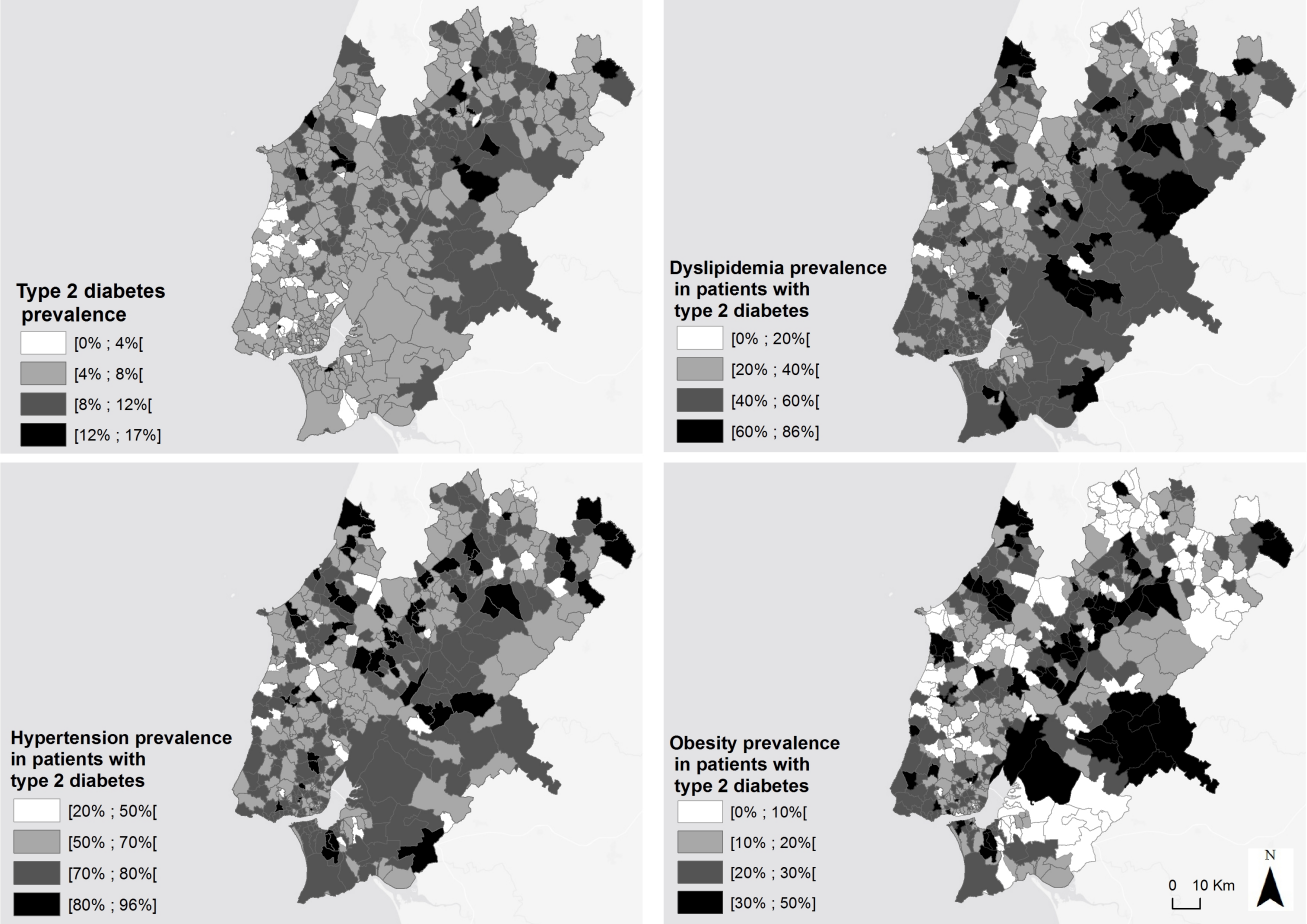
Mapping of diabetes prevalence and comorbiditiesâ€™ distribution, by parish, in Lisbon and Tagus Valley. Image copyright: the authors.

### Geographic Analysis

The maps of prevalence for diabetes, dyslipidemia, hypertension, and obesity showed considerable variation across the region of Lisbon and Tagus Valley, with some parishes showing higher proportions than others (Figure 1). Geographic analysis enabled the identification of high-prevalence areas for diabetes, hypertension, dyslipidemia, and obesity.

## Discussion

### Principal Findings

This study demonstrated the feasibility of collecting, analyzing, and geographically displaying EHR data. Our study showed a prevalence of diagnosed diabetes in primary care similar to previously reported estimates, as well as a high proportion of diagnosed hypertension, consistent with the literature [8]. One way to apply this information would be to focus initial public health efforts in areas where the prevalence of diabetes and comorbidities seems to be higher, analyzing and addressing possible reasons for that discrepancy, at the community-level.

Nevertheless, we found that individual-level data that is extractable from the primary care information systems in Portugal is still limited. We did not have access to data regarding schooling, socio-economic status (SES), diabetes medication, or biometric and lab data. Moreover, ethnicity data is not usually allowed to be collected in Portugal, hampering a comprehensive analysis of health care disparities in ethnic minority groups.

A more accurate and comprehensive GIS analysis was not possible due to lack of access to individual-level zip code of residence information or parish-level data on the social and environmental determinants of health (eg, schooling, SES, housing, walkability, green spaces, distance from grocery stores, fast food chains).

Furthermore, for comprehensive outcomes monitoring to occur, it should be possible to link data from primary care and hospital EHRs, as well as other health institutions (eg, pharmacies, labs) [9]. The integration of these sources of data, in combination with information on the social and environmental determinants of health, would have the potential to render a more complete picture of the health state of communities [2].

Unfortunately, a great amount of data remains siloed in institutions, fragmented, and generally inaccessible to the ones who could bring meaning to it: clinicians, public health workers, researchers, and, most importantly, patients. It is important that health-related data are increasingly treated as a public good and an essential element of a learning health care system.

### Strengths and Limitations

This study had several strengths. It was the first in Portugal to analyze data routinely collected from EHRs, producing small-area maps of the distribution of diabetes and comorbidities, in an entire region. The large sample size and considerable amount of structured data ensure some robustness to the results. The results of our study need to be interpreted in the context of its cross-sectional design. Selection bias cannot be excluded, and two specific groups of individuals might be missing from our sample: people with health care accessibility issues, and people covered by private insurance, who do not normally use the public primary care services.

Finally, it is important to keep in mind that the interpretation of EHR data is generally subject to certain bias (eg, selection, misclassification, surveillance), and should be done skeptically, to distinguish real signals from random noise [10].

### Implications for Clinical Practice, Research, and Health Policy

Given the potential of this approach to improve chronic disease surveillance, awareness should be promoted among policy makers regarding the importance of data access, ownership, security, privacy, quality, and comprehensiveness. Furthermore, buy-in from clinicians should be promoted, and every effort should be made for data entry not to be an extra burden in daily practice. A necessary condition to improve data quality and comprehensiveness is facilitating and streamlining its collection, with clinician-friendly EHRs, and patient involvement in data gathering and integration.

Future studies should explore the effects of small-area characteristics (eg, socioeconomic and environmental factors, health care services availability) on individual health, namely in regions where the burden of diabetes is higher. Analyzing the correlation of health outcomes with the social determinants of health may facilitate the implementation of targeted interventions and an optimal allocation of available resources. Furthermore, by identifying high-risk localities, public health efforts may be able to delineate and prioritize community-based strategies, an important element of the Chronic Care model.

### Conclusion

In summary, primary care EHR data shows potential to be used in public health surveillance of chronic diseases, in particular with the help of GIS. Clinical data routinely collected in daily practice, when combined with information on the social and environmental determinants of health, has the potential to render a more complete picture of the health state of communities.

## Abbreviations

EHRs: electronic health records
GIS: geographic information systems
ICPC: International Classification of Primary Care
SES: socio-economic status
